# Combination therapy with 3% diquafosol tetrasodium ophthalmic solution and sodium hyaluronate: an effective therapy for patients with dry eye after femtosecond laser-assisted *in situ* keratomileusis

**DOI:** 10.3389/fmed.2023.1160499

**Published:** 2023-04-20

**Authors:** Tianjiao Wang, Yu Di, Ying Li

**Affiliations:** ^1^4+4 Medical Doctor Program, Chinese Academy of Medical Sciences, Peking Union Medical College, Beijing, China; ^2^Department of Ophthalmology, Peking Union Medical College Hospital, Chinese Academy of Medical Sciences, Beijing, China

**Keywords:** dry eye, LASIK, diquafosol tetrasodium, mucin, lipid layer, corneal nerve

## Abstract

**Purpose:**

To assess the effect of combination therapy with 3% diquafosol tetrasodium (DQS) and sodium hyaluronate (HA) for dry eye after femtosecond laser-assisted *in situ* keratomileusis (FS-LASIK).

**Design:**

Prospective nonrandomized comparative trial.

**Methods:**

The prospective study included 80 eyes of 40 patients who underwent FS-LASIK with or without preoperative dry eye. Patients were divided into a combination group and a HA group according to their willingness and the doctor’s advice. The combination group was treated with DQS six times a day and HA four times a day, and the HA group was treated with HA four times a day after FS-LASIK. Ocular surface disease index (OSDI), ocular symptom score, vision-related score, environmental score, tear meniscus height (TMH), first non-invasive tear breakup time (NIBUT-First), average non-invasive tear breakup time (NIBUT-Ave), tear breakup time (TBUT), Schirmer I test (SIT), corneal fluorescein staining score (CFS), bulbar redness score, limbal redness score, lipid layer grade (LLG), meiboscore, lid margin abnormality, corneal sensitivity, and corneal nerve parameters were examined before surgery and at 1 week and 1 month after surgery. Surface regularity index (SRI) was also examined before surgery and at 1 month postoperatively.

**Results:**

OSDI score (*p* = 0.024) and vision-related score (*p* = 0.026) were significantly lower in the combination group than in the HA group at 1 month after FS-LASIK, especially in patients with preoperative dry eye symptoms. The increasements of CFS (*p* = 0.018), bulbar redness score (*p* = 0.021), and limbal redness score (*p* = 0.009) were significantly lower in the combination group than in the HA group at 1 week after FS-LASIK. But other ocular surface parameters showed no difference between both groups at 1 week and 1 month after FS-LASIK. LLG was significantly higher in the combination group than in the HA group at 1 week (*p* = 0.004) and 1 month (*p* < 0.001) after surgery, especially in patients with high meiboscore. Additional DQS significantly improved corneal sensitivity in patients without preoperative dry eye symptoms at 1 month after FS-LASIK (*p* = 0.041).

**Conclusion:**

The combination therapy with DQS and HA significantly relieved subjective symptoms, improved ocular surface status, and had the potential to promote corneal nerve growth in patients after FS-LASIK.

## 1. Introduction

Femtosecond laser-assisted *in situ* keratomileusis (FS-LASIK) is one of the mainstream corneal refractive procedures. With the improvement of surgical quality, the incidence of severe complications of FS-LASIK has gradually decreased. However, dry eye disease (DED) is still one of the most common complications after FS-LASIK (1). The pathophysiology mechanism of post FS-LASIK dry eye is mainly associated with damage to corneal nerve fibers and ocular surface tissues (1). Most dry eye symptoms disappear within 6–9 months after surgery as corneal nerve regenerates (2, 3). However, we should be aware that patients may still suffer from dry eye in the recovery period. Therefore, treating postoperative dry eye is necessary to improve patient satisfaction. At the same time, under the influence of widespread use of video display terminals (4), frequent contact lens wear (5), sleep deprivation (6), and so on, the number of patients with dry eye symptoms or signs before refractive surgery has increased dramatically. Preexisting dry eye is a high risk factor for postoperative dry eye (1), bringing a new challenge to the treatment of dry eye after refractive surgery.

Sodium hyaluronate (HA) is the first-line therapy for post-LASIK dry eye and alleviates DED symptoms temporarily by virtue of water retentive property (7). However, considering its therapeutic mechanism is single, it may not be effective enough in treating severe or prolonged dry eyes. Other treatments for dry eye are also not perfect. Long-term use of glucocorticoids can produce side effects such as high intraocular pressure and cataract (8). Cyclosporine is irritating for part of patients (9). Autologous serum must be preserved under strict conditions, which is inconvenient for patients (10). Punctal plug leads to complications such as epiphora and suppurative canaliculitis (11).

As recently reported, 3% diquafosol tetrasodium ophthalmic solution (Diquas^®^, DQS), a P2Y2 receptor agonist that acts on goblet cells, corneal and conjunctival epithelium, and meibomian glands, can promote tear fluid, mucin, and lipid secretion and help epithelium repair (12, 13). Several clinical trials have shown that DQS significantly reduces corneal and conjunctival staining, prolongs tear breakup time (TBUT), increases Schirmer test score and improves subjective symptoms in patients with dry eye (14–17). For post-LASIK dry eye, several studies have reported that DQS alone or combined with HA can improve part of subjective symptoms and objective signs and that additional DQS may be helpful in postoperative near and distance visual acuity (18, 19). However, the aforementioned studies did not assess the effects of DQS on the lipid layer, meibomian glands and corneal nerve, which were also involved in the mechanism of post-LASIK dry eye.

This prospective study aimed to evaluate the effect of combination therapy with 3% diquafosol ophthalmic solution and sodium hyaluronate ophthalmic solution eye drops on subjective symptoms, objective signs, meibomian glands, and corneal nerve.

## 2. Methods

This prospective cohort trial assessed the efficacy of combination therapy with DQS and HA in terms of subjective symptoms, ocular surface parameters, surface regularity index, meibomian gland parameters, and corneal nerve parameters after FS-LASIK. The study followed the tenets of the Declaration of Helsinki and was approved by the Ethics Committee of Peking Union Medical College Hospital. Informed consent was obtained from all subjects. All patients in the study were enrolled from Ophthalmology Department of Peking Union Medical College Hospital from August 2021 to June 2022.

### 2.1. Patients

The study was designed to enroll patients who were willing to undergo FS-LASIK and met the indications. The exclusion criteria for the participants included progressive myopia or astigmatism, strabismus or hyperopia, history of ocular trauma or eye surgery, diagnosed autoimmune disease or connective tissue disease, ocular abnormalities or diseases such as fundus lesions, cataract and glaucoma, unwillingness to cooperate with the study, and postoperative use of eye drops for dry eye except for DQS and HA. Participants were assigned into a combination group and a HA group according to their willingness and the doctor’s advice. The combination group used 3% diquafosol tetrasodium ophthalmic solution (Diquas^®^; Santen Pharmaceutical Co, Ltd., Osaka, Japan) six times a day and 0.1% sodium hyaluronate ophthalmic solution (Hylo-Comod^®^, Ursapharm, Saarbrucken, Germany) four times a day, while the HA group used only 0.1% sodium hyaluronate ophthalmic solution (Hylo-Comod^®^, Ursapharm, Saarbrucken, Germany). Both groups used eye drops from postoperative day 1 to postoperative 1 month after FS-LASIK.

### 2.2. Preoperative and postoperative assessments

Except for routine preoperative and postoperative examinations for FS-LASIK such as slit-lamp examination, all patients underwent comprehensive dry eye examinations preoperatively and at 1 week and 1 month after FS-LASIK. The following parameters of both eyes were assessed at each visit: ocular surface disease index (OSDI), tear meniscus height (TMH), first non-invasive tear film break-up time (NIBUT-First), average non-invasive tear film break-up time (NIBUT-Ave), tear breakup time, Schirmer I test score (SIT), corneal fluorescein staining score (CFS), bulbar redness score, limbal redness score, lipid layer grade (LLG), meiboscore, lid margin abnormality, corneal sensitivity, and corneal nerve parameters. Surface regularity index (SRI) was measured by corneal tomography (Tomey TMS-4; Tomey, Nagoya, Japan) before surgery and at 1 month after surgery.

Subjective symptoms were evaluated by OSDI questionnaire. OSDI score was calculated as follows: OSDI = (sum of scores for all questions answered ×25)/(total number of answered questions). The OSDI questionnaire consists of 12 questions and 3 subscales including the ocular symptoms (questions 1–5), the vision-related function (questions 6–9), and the environmental triggers (questions 10–12) (20). The scores of the three parts were calculated in the same way as OSDI score. According to the study of Miller et al. (21), patients were divided into non-dry eye group (OSDI <13 points) and dry eye group (OSDI ≥13 points) according to OSDI score.

TMH, NIBUT-First, NIBUT-Ave, LLG, meiboscore, lid margin abnormality, bulbar and limbal redness scores were measured by DED-1L dry eye analyzer (Chongqing Kanghua Ruiming Technology Co., LTD). TMH below the center of the pupil was measured by DED analyzer. Patients were requested to refrain from blinking and then NIBUT-First and NIBUT-Ave were measured automatically by DED analyzer. The lipid layer was graded by observing the interference of color and comparing it to the examples in DED analyzer. The correlation between lipid layer grade and thickness was as follows: grade 1: <15 nm, grade 2: ≈15 nm, grade 3: ≈30 nm, grade 4: ≈30-80 nm, grade 5: ≈80 nm, grade 6: ≈80-120 nm, grade 7: ≈120-160 nm. Meiboscore was defined by the ratio of meibomian gland loss: 0 points: no or minimal MG loss, 1 point: ≤1/3 MG loss, 2 points: 1/3–2/3 MG loss, 3 points: >2/3 MG loss (22). Both upper and lower eyelids were measured, and the total meiboscore was calculated as the sum of the upper and lower lid meiboscores. Patients were divided into two groups: low meiboscore group (both upper and lower lid meiboscores<2) and high meiboscore group (upper lid meiboscore≥2 or lower lid meiboscore≥2). Lid margin abnormality was evaluated as follows: grade 1: clear and transparent lid plugs; grade 2: meibomian gland orifices cap crown and protrusion; grade 3: lipid plugs at gland orifices, loss of lid margin mucosa, and hyperkeratosis of orifices; grade 4: irregular lid margin, loss of lid gland orifice, thickened posterior lid margin, and neovascularization.

The cornea was stained with a single-use fluorescein strip wetted with one drop of tobramycin eye drops. TBUT was the time interval between blinking and the appearance of the first dry spot, and the average of the three repeated measurements was recorded. CFS was evaluated by 12-point method: the cornea was divided into four quadrants and each quadrant was scored individually, 0 points: no staining; 1 point: mild staining with a few scattered dots of stains; 2 points: moderate staining between 1 and 3; 3 points: severe staining with confluent stains or corneal filaments, and the total of the four quadrant scores represented CFS (23). Schirmer test without anesthesia was performed by placing a 5 mm × 35 mm Schirmer paper strip into the temporal one-third of the lower conjunctival sac and measuring the length of the wet paper strip after keeping the eyes closed for 5 min.

Central corneal sensitivity was measured by a Cochet–Bonnet corneal esthesiometer as previously described (24). *In vivo* confocal microscopy (IVCM, Heidelberg Retina Tomograph III Rostock Cornea Module, Heidelberg, Germany) was used to observe the corneal subbasal nerve plexus, and images of 384 × 384 pixels in the range of 400 × 400 μm could be acquired. One drop of 0.4% oxybuprocaine hydrochloride eye drops was applied to each eye of the patients. The focal length of IVCM was adjusted to Bowman’s membrane to observe the corneal subbasal nerve plexus, and three to five clearest and most representative images were selected for measurement. As reported previously, a software program ACCMetrics designed by the University of Manchester Research Group (Manchester, United Kingdom) can automatically analyze the subbasal corneal nerve images with better consistency than manual measurements (25, 26). The following parameters were measured: nerve fiber density (CNFD): the number of nerve fibers per mm^2^; nerve branch density (CNBD): the number of branch points on the main fiber per mm^2^; nerve fiber length (CNFL): the total length of nerve fibers per mm^2^ (mm/mm^2^); nerve fiber total branch density (CTBD): the total number of branch points per mm^2^; nerve fiber area (CNFA): the total nerve fiber area per mm^2^ (mm^2^/mm^2^); nerve fiber width (CNFW): the average width of nerve fibers per mm^2^ (mm/mm^2^); corneal nerve fractal dimension (CNFrD): a parameter to measure the structural complexity of corneal nerves.

### 2.3. Surgical technique

In FS-LASIK, the Visual Max femtosecond laser system (Carl Zeiss Meditec, Jena, Germany) was used to create a corneal stromal flap with a flap thickness of 90–110 mm and a flap diameter of 8.1 mm. And laser ablation was performed using the WaveLight EX500 excimer laser (Alcon Laboratories Inc.) with an optical zone diameter of 6.0–6.5 mm. The balanced salt solution was used to flush the stromal bed and then the flap was repositioned. In addition to DQS and HA, all patients were treated postoperatively with 0.5% Loteprednol etabonate ophthalmic suspension (Lotemax Bausch & Lomb Incorporated) in tapering dosages for 4 weeks (starting with four times per day).

### 2.4. Statistical analysis

All statistical analyses were performed using SPSS 25.0. The normality of data distribution was tested using the Shapiro–Wilk test. Descriptive parameters were expressed as mean ± standard deviation (SD). Normally distributed parameters between the combination group and the HA group were compared using independent samples *t*-test, whereas the non-normally distributed parameters were compared using Mann–Whitney *U* test. One-way repeated measures ANOVA was used to compare the normally distributed parameters for time points in each group, and Friedman test was used to compare the non-normally distributed parameters. For the normally distributed parameters, the bivariate correlation analysis was performed using the Pearson correlation analysis, and if else using the Spearman correlation analysis. When it came to the analysis of the correlation between OSDI score and other parameters, one eye of each patient was included using a random number table. In the rest of the statistical analyses, both eyes of the patients were included. A *p* value <0.05 was considered statically significant.

## 3. Results

This study included 40 eyes of 20 patients in each group. There were no significant differences in age (30.8 ± 7.4, 32.1 ± 6.8 years for the combination group, HA group, respectively), and sex (16/4, 16/4, female/male, respectively) between the two groups before FS-LASIK. And subjective symptom parameters, ocular surface parameters, meibomian gland parameters and corneal nerve parameters were not significantly different between the two groups at the preoperative visit. The preoperative and postoperative parameters for the two groups are summarized in Table 1. The postoperative alterations of each parameter for the two groups are presented in Supplementary Table S1.

**Table 1:** Changes in dry eye parameters, meibomian gland parameters and corneal nerve parameters in the combination group and the HA group.

	Preop			1 Week			1 Month		
	DQS + HA	HA	*P*	DQS + HA	HA	*P*	DQS + HA	HA	*P*
Subjective symptoms									
OSDI	17.55 ± 15.70	18.39 ± 17.31	0.841	27.07 ± 18.34^‡^	38.72 ± 24.13^†††^	0.174	16.97 ± 9.96	28.72 ± 19.65^†^	0.024^*^
Ocular symptom score	14.25 ± 11.15	19.75 ± 18.17	0.414	27.50 ± 17.81^‡‡^	39.00 ± 23.65^†††^	0.165	18.25 ± 13.11	29.00 ± 20.43	0.091
Vision-related score	23.33 ± 32.87	16.56 ± 25.25	0.529	28.13 ± 23.07	44.38 ± 32.72^††^	0.157	13.75 ± 12.60	30.83 ± 25.21^†^	0.026^*^
Environmental score	15.00 ± 14.71	19.17 ± 17.33	0.414	24.12 ± 19.47	31.14 ± 25.21	0.644	19.17 ± 12.35	25.83 ± 20.93	0.398
Ocular surface parameters									
TMH (mm)	0.26 ± 0.13	0.23 ± 0.08	0.950	0.24 ± 0.09	0.22 ± 0.07	0.544	0.26 ± 0.09	0.24 ± 0.10	0.209
SIT (mm)	13.0 ± 9.5	17.3 ± 11.2	0.129	8.8 ± 7.2^‡^	12.6 ± 11.3^††^	0.267	10.8 ± 9.2	12.9 ± 10.7^†^	0.553
CFS	1.0 ± 1.8	0.7 ± 1.2	0.623	1.5 ± 1.8	2.2 ± 1.7^††^	0.052	1.6 ± 1.9	1.8 ± 2.0^††^	0.637
NIBUT-First (s)	8.32 ± 4.99	8.99 ± 7.09	0.840	9.02 ± 8.18	7.66 ± 7.12	0.482	6.53 ± 4.95	7.69 ± 4.87	0.195
NIBUT-Ave (s)	11.90 ± 4.84	12.21 ± 6.69	0.847	11.88 ± 7.27	11.71 ± 6.81	0.939	10.54 ± 6.12	11.23 ± 5.84	0.560
TBUT (s)	5.1 ± 4.3	5.0 ± 3.5	0.459	4.3 ± 3.0	4.5 ± 3.3	0.850	3.8 ± 1.9	3.8 ± 2.5	0.442
Bulbar redness score	1.31 ± 0.29	1.24 ± 0.17	0.802	1.25 ± 0.25	1.27 ± 0.18	0.368	1.28 ± 0.25	1.27 ± 0.19	0.844
Limbal redness score	1.19 ± 0.33	1.13 ± 0.19	0.825	1.10 ± 0.27^‡^	1.12 ± 0.15	0.055	1.16 ± 0.25	1.15 ± 0.14	0.600
SRI	0.15 ± 0.13	0.13 ± 0.13	0.505	–	–	–	0.28 ± 0.20^‡‡^	0.29 ± 0.22^†††^	0.866
Meibomian gland parameters									
Lipid layer grade	4.5 ± 1.3	4.3 ± 1.3	0.514	4.2 ± 1.1	3.4 ± 1.2^††^	0.004^**^	4.8 ± 0.9	3.4 ± 1.3^†^	<0.001^***^
Meiboscore	2.8 ± 1.6	3.0 ± 1.2	0.684	2.9 ± 1.6	3.0 ± 1.1	0.661	3.0 ± 1.6	3.1 ± 1.1	0.734
Lid margin abnormality	2.5 ± 0.7	2.3 ± 0.5	0.461	2.5 ± 0.6	2.4 ± 0.6	0.932	2.6 ± 0.7	2.6 ± 0.8	0.771
Corneal nerve parameter									
Corneal sensitivity (mm)	58.4 ± 3.5	58.3 ± 3.7	0.958	12.5 ± 18.5^‡‡‡^	10.9 ± 14.5^†††^	0.878	20.5 ± 19.7^‡‡‡^	16.5 ± 18.0^†††^	0.543
CNFD (/mm^2^)	20.0 ± 7.4	19.9 ± 7.2	0.940	0.6 ± 1.8^‡‡‡^	0.2 ± 0.7^†††^	0.134	0.8 ± 2.1^‡‡‡^	0.1 ± 0.5^†††^	0.038^*^
CNBD (/mm^2^)	24.8 ± 14.3	21.4 ± 15.8	0.167	0.3 ± 1.2^‡‡‡^	0.1 ± 0.7^†††^	0.308	0.6 ± 1.6^‡‡‡^	0.2 ± 1.0^†††^	0.098
CNFL (mm/mm^2^)	12.9 ± 3.0	12.6 ± 2.9	0.610	2.5 ± 1.3^‡‡‡^	2.2 ± 1.0^†††^	0.322	2.4 ± 1.6^‡‡‡^	2.0 ± 1.0^†††^	0.683
CTBD (/mm^2^)	38.8 ± 18.8	35.9 ± 21.9	0.312	6.5 ± 6.7^‡‡‡^	3.8 ± 4.1^†††^	0.065	5.0 ± 5.1^‡‡‡^	3.4 ± 3.3^†††^	0.300
CNFA (mm^2^/mm^2^)	0.0058 ± 0.0016	0.0054 ± 0.0020	0.167	0.0019 ± 0.0011^‡‡‡^	0.0018 ± 0.0009^†††^	0.732	0.0018 ± 0.0011^‡‡‡^	0.0016 ± 0.0007^†††^	0.538
CNFW (mm/mm^2^)	0.021 ± 0.001	0.021 ± 0.001	0.603	0.027 ± 0.006^‡‡‡^	0.029 ± 0.003^†††^	0.153	0.027 ± 0.004^‡‡‡^	0.028 ± 0.003^†††^	0.199
CNFrD	1.47 ± 0.04	1.46 ± 0.03	0.516	1.22 ± 0.11^‡‡‡^	1.22 ± 0.66^†††^	0.408	1.22 ± 0.09^‡‡‡^	1.21 ± 0.06^†††^	0.684

Data are presented as means ± standard deviation. Preop, preoperative; OSDI, ocular surface disease index; TMH, tear meniscus height; SIT, Schirmer I test; CFS, corneal fluorescein staining score; NIBUT-First, first non-invasive tear breakup time; NIBUT-Ave, average non-invasive tear breakup time; TBUT, tear breakup time; SRI, surface regularity index; CNFD, nerve fiber density; CNBD, nerve branch density; CNFL, nerve fiber length; CTBD, nerve fiber total branch density; CNFA, nerve fiber area; CNFW, nerve fiber width; CNFrD, nerve fiber fractal dimension. ^*^p < 0.05, ^**^p < 0.01, and ^***^p < 0.001 between the combination group and the HA group by independent samples *t*-test or Mann–Whitney *U* test. ^‡^*p* < 0.05, ^‡‡^*p* < 0.01, ^‡‡‡^*p* < 0.001 between preoperative visit and postoperative visits in the combination group and ^†^*p* < 0.05, ^††^*p* < 0.01, ^†††^*p* < 0.001 between preoperative visit and postoperative visits in the HA group by one-way repeated measures ANOVA or Friedman test. DQS, diquafosol tetrasodium; HA, sodium hyaluronate; DQS + HA, combination of diquafosol tetrasodium and sodium hyaluronate.

### 3.1. Subjective symptoms

**Figure 1:**
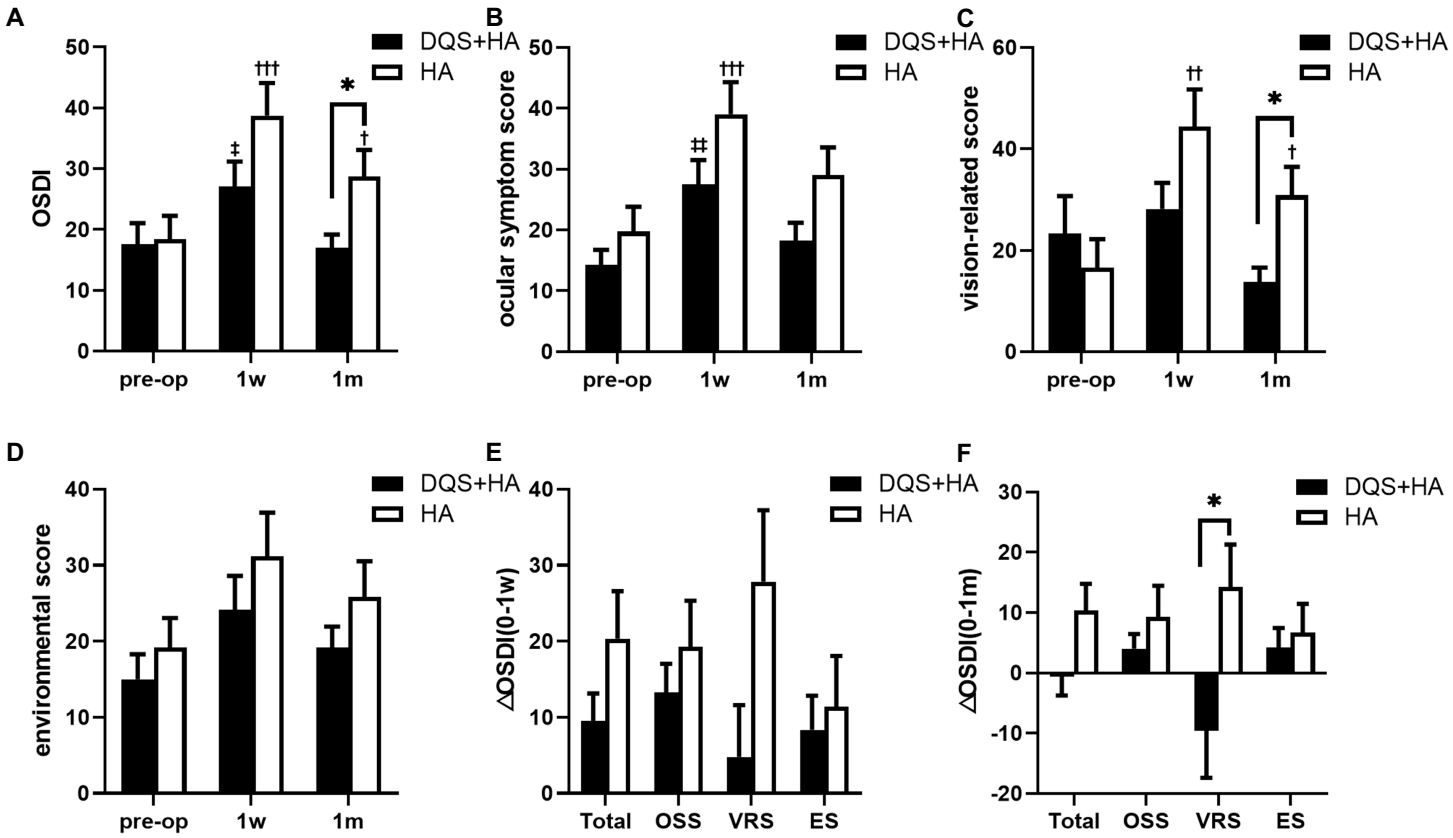
Changes in OSDI score **(A)**, ocular symptom score **(B)**, vision-related score **(C)**, and environmental score **(D)** in patients after FS-LASIK and the alterations from preoperative to postoperative 1 week **(E)** and postoperative 1 month **(F)**. Mean value ± standard error. *p < 0.05 between the combination group and the HA group by independent samples *t*-test or Mann–Whitney *U* test. ^‡^*p* < 0.05, ^‡‡^*p* < 0.01 between preoperative visit and postoperative visits in the combination group and ^†^*p* < 0.05, ^††^*p* < 0.01, ^†††^*p* < 0.001 between preoperative visit and postoperative visits in the HA group by one-way repeated measures ANOVA or Friedman test. OSDI, ocular surface disease index; OSS, ocular symptom score; VRS, vision-related score; ES, environmental score; DQS + HA, combination of diquafosol tetrasodium and sodium hyaluronate; HA, sodium hyaluronate.

Figure 1 represents the changes in subjective symptom parameters in both groups. OSDI score for the combination group and ocular symptom score for both groups showed a transient increase at postoperative 1 week (OSDI for combination group, *p* = 0.013; ocular symptom score for combination group, *p* = 0.001; ocular symptom score for HA group, *p* < 0.001). And there were significant increases in OSDI score and vision-related score for the HA group across all study periods (*p* < 0.001, *p* = 0.004 for OSDI score and ocular symptom score at postoperative 1 week, respectively; *p* = 0.027, *p* = 0.043 for OSDI score and vision-related score at postoperative 1 month, respectively). Vision-related score for the combination group and environmental score for both groups did not change from the preoperative to postoperative periods. No significant differences in subjective symptom parameters were observed between the two groups at postoperative 1 week, but OSDI score and vision-related score for the combination group were significantly lower than those for the HA group at 1 month after surgery (*p* = 0.024, *p* = 0.026, respectively). Additionally, the increase in vision-related score was significantly lower in the combination group than that in the HA group at 1 month after surgery (*p* = 0.017).

### 3.2. Ocular surface parameters

**Figure 2:**
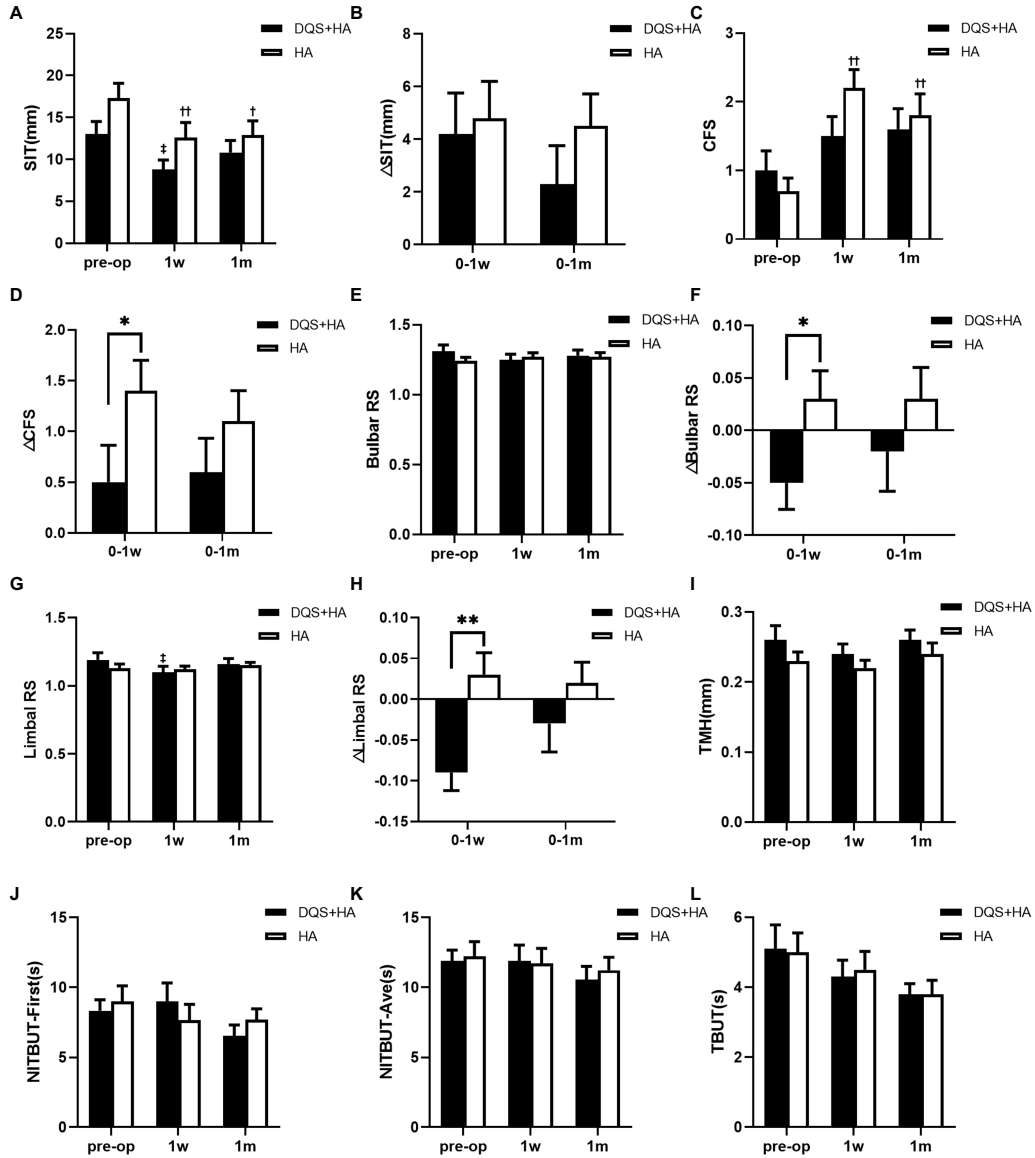
Changes in SIT **(A,B)**, CFS **(C,D)**, bulbar RS **(E,F)**, limbal RS **(G,H)**, TMH **(I)**, NIBUT-First **(J)**, NIBUT-Ave **(K)**, and TBUT **(L)** in patients after FS-LASIK. Mean value ± standard error. *p < 0.05 and **p < 0.01 between the combination group and the HA group by independent samples *t*-test or Mann–Whitney *U* test. ^‡^*p* < 0.05 between preoperative visit and postoperative visits in the combination group and ^†^*p* < 0.05, ^††^*p* < 0.01 between preoperative visit and postoperative visits in the HA group by one-way repeated measures ANOVA or Friedman test. SIT, Schirmer I test; CFS, corneal fluorescein staining score; bulbar RS, bulbar redness score; limbal RS, limbal redness score; TMH, tear meniscus height; NIBUT-First, first non-invasive tear breakup time; NIBUT-Ave, average non-invasive tear breakup time; TBUT, tear breakup time; DQS + HA, combination of diquafosol tetrasodium and sodium hyaluronate; HA, sodium hyaluronate.

SIT score revealed a significant decrease in both groups at 1 week after FS-LASIK compared with the preoperative score (combination group, *p* = 0.036; HA group, *p* = 0.001). However, only in the HA group SIT score remained significantly lower than before at postoperative 1 month (*p* = 0.036). There was no significant difference between the combination group and the HA group at any time point (Figure 2A). The decrease in SIT score for the combination group tended to be lower than that for the HA group but not significantly (Figure 2B).

CFS score for the combination group showed no significant difference between the preoperative and postoperative visits. On the contrary, CFS score for the HA group significantly increased at postoperative visits compared with the preoperative value (*p* = 0.001 at postoperative 1 week, *p* = 0.009 at postoperative 1 month) (Figure 2C). Though there was no significant difference between two groups in CFS score across all study periods, the increasement of the score for the combination group was significantly lower than that for the HA group at 1 week after FS-LASIK (*p* = 0.018), and the similar trend was observed at 1 month after FS-LASIK (Figure 2D).

Regarding bulbar and limbal redness scores, only the limbal redness score significantly decreased in the combination group at 1 week postoperatively (*p* = 0.018). And the decreases in redness scores in the combination group were significantly higher than those in the HA group at 1 week after FS-LASIK (bulbar redness score, *p* = 0.021; limbal redness score, *p* = 0.009) (Figures 2E–H).

There were no significant differences in TMH, NIBUT-First and NIBUT-Ave between two groups across all study periods. And these values did not change in both groups after surgery (Figures 2I–L).

SRI decreased significantly in both groups at 1 month after FS-LASIK compared with the preoperative values (combination group, *p* = 0.003; HA group, *p* < 0.001), but no significant difference was observed between two groups before and after FS-LASIK.

### 3.3. Meibomian gland parameters

**Figure 3:**
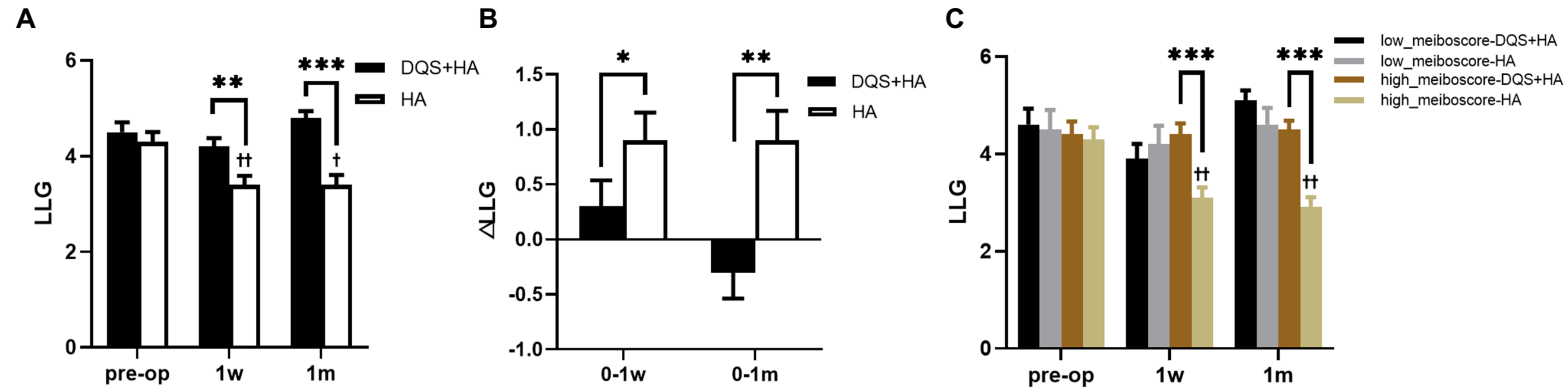
Changes in LLG in patients after FS-LASIK **(A,B)** and in different meiboscore subgroups **(C)**. Mean value±standard error. The sample sizes of low meiboscore-DQS + HA group, low meiboscore-HA group, high meiboscore-DQS + HA group, and high meiboscore-HA group were 16, 12, 24, and 28 eyes, respectively. *p < 0.05, **p < 0.01, and ***p < 0.001 between the combination group and the HA group by Mann–Whitney *U* test. ^†^*p* < 0.05 and ^††^*p* < 0.01 between preoperative visit and postoperative visits in the HA group by Friedman test. LLG, lipid layer grade; DQS, diquafosol tetrasodium; HA, sodium hyaluronate.

LLG significantly reduced in the HA group at the follow-up time (*p* = 0.003 at postoperative 1 week, *p* = 0.030 at postoperative 1 month), but it did not change in the combination group. LLG was significantly better in the combination group than that in the HA group after surgery (*p* = 0.004 at postoperative 1 week, *p* < 0.001 at postoperative 1 month) (Figures 3A,B). Meiboscore and Lid margin abnormality were not significantly different between two groups, and were unchanged in both groups after FS-LASIK.

### 3.4. Corneal nerve parameters

Corneal sensitivity, CNFD, CNBD, CNFL, CTBD, CNFA, CNFW, and CNFrD significantly decreased in both groups after FS-LASIK compared with the preoperative values (all *p* < 0.001). There were no significant differences in corneal sensitivity and corneal nerve parameters between two groups at the follow-up time, except that CNFD in the combination group was significantly higher than that in the HA group at 1 month postoperatively (*p* = 0.038) (Table 1). But the significance disappeared in the alteration of CNFD (Supplementary Table S1).

### 3.5. Subgroup analysis

Correlation coefficients were calculated to explore the effects of preoperative clinical parameters on OSDI score and LLG at postoperative 1 month (Table 2). Postoperative OSDI score positively correlated to preoperative OSDI score. Postoperative LLG negatively correlated to preoperative limber redness score and meiboscore, and positively correlated to preoperative corneal sensitivity.

**Table 2:** Correlation coefficients between preoperative parameters and OSDI and LLG at postoperative 1 month in all patients.

	Postop 1 m-OSDI		Postop 1 m-LLG	
	Correlation coefficient	*p*	Correlation coefficient	*p*
Age (year)	0.098	0.548	0.054	0.740
Spherical equivalent (D)	−0.088	0.590	−0.068	0.548
Preop OSDI	0.492	0.001^**^	−0.059	0.717
Preop TMH (mm)	−0.212	0.188	0.044	0.697
Preop SIT (mm)	0.019	0.907	−0.137	0.224
Preop CFS	0.043	0.792	0.037	0.743
Preop NIBUT-First (s)	−0.121	0.455	0.047	0.679
Preop NIBUT-Ave (s)	−0.113	0.486	0.055	0.626
Preop TBUT (s)	0.160	0.323	0.056	0.623
Preop bulbar redness score	0.026	0.874	0.070	0.537
Preop limbal redness score	−0.057	0.727	−0.349	0.001^**^
Preop LLG	−0.103	0.527	0.145	0.199
Preop meiboscore	−0.026	0.874	−0.330	0.003^**^
Preop lid margin abnormality	0.024	0.885	0.020	0.858
Preop SRI	0.233	0.149	−0.050	0.662
Preop corneal sensitivity (mm)	0.175	0.281	0.232	0.038^*^
Preop CNFD (/mm^2^)	0.031	0.849	−0.029	0.799
Preop CNBD (/mm^2^)	−0.056	0.730	0.004	0.973
Preop CNFL (mm/mm^2^)	−0.041	0.802	−0.065	0.566
Preop CTBD (mm/mm^2^)	−0.116	0.477	−0.034	0.767
Preop CNFA (mm^2^/mm^2^)	−0.176	0.278	−0.001	0.991
Preop CNFW (mm/mm^2^)	0.016	0.923	0.095	0.400
Preop CNFrD	−0.004	0.981	−0.069	0.544

Preop, preoperative; OSDI, ocular surface disease index; LLG, lipid layer grade; TMH, tear meniscus height; SIT, Schirmer I test; CFS, corneal fluorescein staining score; NIBUT-First, first non-invasive tear breakup time; NIBUT-Ave, average non-invasive tear breakup time; TBUT, tear breakup time; SRI, surface regularity index; CNFD, nerve fiber density; CNBD, nerve branch density; CNFL, nerve fiber length; CTBD, nerve fiber total branch density; CNFA, nerve fiber area; CNFW, nerve fiber width; CNFrD, nerve Fiber Fractal Dimension. ^*^p < 0.05 and ^**^p < 0.01 by Pearson correlation analysis or Spearman correlation analysis.

**Figure 4:**
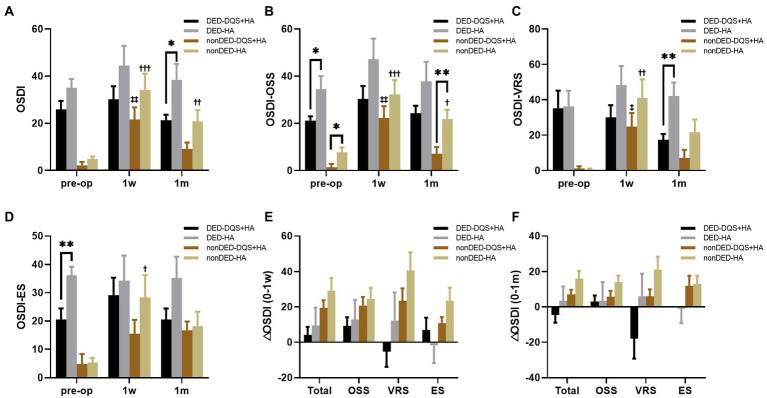
Changes in OSDI score **(A)**, OSS **(B)**, VS **(C)**, ES **(D)** after FS-LASIK and the alterations from preoperative to postoperative 1 week **(E)** and postoperative 1 month **(F)** in dry eye patients and non-dry eye patients. Mean value ± standard error. The sample sizes of DED-DQS + HA group, DED-HA group, nonDED-DQS + HA group, and nonDED-HA group were 13, 9, 7, and 11 patients, respectively. *p < 0.05 and **p < 0.01 between the combination group and the HA group in each subgroup by independent samples *t*-test or Mann–Whitney *U* test. ^‡^*p* < 0.05, ^‡‡^*p* < 0.01 between preoperative visit and postoperative visits in the combination group and ^†^*p* < 0.05, ^††^*p* < 0.01, ^†††^*p* < 0.001 between preoperative visit and postoperative visits in the HA group by one-way repeated measures ANOVA or Friedman test. OSDI, ocular surface disease index; OSS, ocular symptom score; VRS, vision-related score; ES, environmental score; DED, dry eye subgroup; non-DED, non-dry eye subgroup; DQS + HA, combination of diquafosol tetrasodium and sodium hyaluronate; HA, sodium hyaluronate.

For the subgroup divided by preoperative OSDI score, there were no significant differences in preoperative subjective symptom parameters between the combination group and HA group in each subgroup except ocular symptom score and environmental score. For the dry eye subgroup, regardless of whether DQS was used, the subjective symptom parameters did not change significantly after FS-LASIK. However, OSDI score and vision-related score for the combination group were significantly lower than those for the HA group at 1 month postoperatively (*p* = 0.012, *p* = 0.003, respectively). For non-dry eye subgroup, environmental score at 1 week after FS-LASIK and all parameters at 1 month after FS-LASIK showed no significant changes from the preoperative values in the combination group, while in the HA group, all parameters at 1 week after FS-LASIK and OSDI score and vision-related score at 1 month after FS-LASIK significantly increased compared with the preoperative values (*p* < 0.001, *p* < 0.001, *p* = 0.002, *p* = 0.011 for OSDI score, ocular symptom score, vision-related score, and environmental score at postoperative 1 week, respectively; *p* = 0.009, *p* = 0.032 for OSDI score and vision-related score at postoperative 1 month, respectively). And for non-dry eye subgroup, there were no significant differences in subjective symptom parameters between the combination group and the HA group. In each subgroup, it was found that the increasement of each parameter in the combination group tended to be smaller than that in the HA group within the follow-up period (Figure 4). In addition, additional DQS also played a role in other dry eye parameters. For both subgroups, LLG was significantly better in the combination group than that in the HA group at 1 month after FS-LASIK (non-dry eye group, *p* = 0.006; dry eye group, *p* < 0.001). And for non-dry eye subgroup, the same significant difference was also observed in LLG at postoperative 1 week (*p* = 0.001) (Table 3). Furthermore, for non-dry eye subgroup, corneal sensitivity was significantly better in the combination group than that in the HA group at 1 month after FS-LASIK (26.43 ± 20.80 mm vs. 12.237 ± 14.86 mm, *p* = 0.041), while there was no significant difference at 1 week after FS-LASIK (5.36 ± 4.14 mm vs. 13.41 ± 18.02 mm, *p* = 0.665). The increase in corneal sensitivity from 1 week to 1 month after FS-LASIK was significantly higher in the combination group than that in the HA group (21.07 ± 20.59 mm vs. − 1.1 ± 8.99 mm, *p* < 0.001). For dry eye subgroup, CTBD was significant bigger in the combination group than that in the HA group at postoperative 1 week (5.77 ± 5.84/mm^2^ vs. 2.37 ± 2.94/mm^2^, *p* = 0.037), but there was no significant difference in the reduction of CTBD from preoperative to postoperative 1 week between the two groups (−37.78 ± 17.88/mm^2^ vs. − 32.69 ± 20.26/mm^2^, *p* = 0.173).

**Table 3:** Changes in LLG in dry eye patients and non-dry eye patients after FS-LASIK.

	Non-dry eye subgroup			Dry eye subgroup		
	DQS + HA	HA	*p*	DQS + HA	HA	*p*
Preoperative LLG	4.7 ± 1.3	4.5 ± 1.2	0.451	4.4 ± 1.3	4.2 ± 1.4	0.573
LLG_1w	4.9 ± 0.9	3.5 ± 1.1^†^	0.001^**^	3.9 ± 1.1	3.3 ± 1.4	0.474
LLG_1m	4.8 ± 0.4	3.7 ± 1.3	0.006^**^	4.7 ± 1.1	3.1 ± 1.4	<0.001^***^
ΔLLG (0-1w)	0.1 ± 1.2	−1.0 ± 1.7	0.034^*^	−0.5 ± 1.6	−0.8 ± 1.4	0.436
ΔLLG (0-1 m)	0.1 ± 1.3	−0.8 ± 1.7	0.077	0.4 ± 1.6	−1.1 ± 1.7	0.026^*^
ΔLLG (1w-1 m)	−0.1 ± 0.9	0.2 ± 1.2	0.451	0.9 ± 1.5	−0.2 ± 1.3	0.038^*^

Data are presented as means ± standard deviation. The sample sizes of the four groups were 13, 9, 7, and 11 patients, respectively. LLG, lipid layer grade; LLG_1w, lipid layer grade at postoperative 1 week; LLG_1m, lipid layer grade at postoperative 1 month; ΔLLG (0-1w), the alteration of LLG from preoperative to postoperative 1 week; ΔLLG (0-1 m), the alteration of LLG from preoperative to postoperative 1 month; ΔLLG (1w-1 m), the alteration of LLG from postoperative 1 week to postoperative 1 month. ^*^p < 0.05, ^**^p < 0.01, and ^***^p < 0.001 between the combination group and the HA group by independent samples *t*-test or Mann–Whitney *U* test. ^†^*p* < 0.05 between preoperative visit and postoperative visits in the HA group by one-way repeated measures ANOVA or Friedman test. DQS, diquafosol tetrasodium; HA, sodium hyaluronate; DQS + HA, combination of diquafosol tetrasodium and sodium hyaluronate.

According to preoperative LLG, patients were divided into high meiboscore subgroup and low meiboscore subgroup (Supplementary Table S2). There were no significant differences in LLG between the combination group and the HA group in each subgroup before surgery. Postoperative LLG of the HA group in the high meiboscore subgroup significantly decreased compared with preoperative LLG (*p* = 0.002 at postoperative 1 week, *p* = 0.003 at postoperative 1 month), while no changes were seen in the other groups. In the high meiboscore subgroup, LLG was significantly higher in the combination group than that in the HA group (both *p* < 0.001), but there was no difference in the low meiboscore subgroup (Figure 3C).

## 4. Discussion

HA may not be sufficient to cure post-LASIK dry eye due to the rise in patients with preoperative dry eye or underlying dry eye symptoms. The present study found that the combination therapy with DQS and HA after FS-LASIK significantly improved the postoperative subjective symptoms, ocular surface status, and lipid layer thickness and possibly promote corneal nerve regeneration compared with HA monotherapy.

Several factors are involved in the pathophysiological mechanism of dry eye after FS-LASIK (1). First, loss of corneal innervation after FA-LASIK leads to decreased corneal sensitivity, which in turn affects the corneal-lacrimal gland and corneal reflex. Second, mucin secretion is downregulated in the hyponeurotrophic state, which affects tear film stability. Third, the surgery itself disrupts goblet cells and increases ocular surface irregularities, further reducing tear film stability. Some previous studies revealed that not only dry eye symptoms and OSDI score (27, 28) but also objective symptoms, such as TBUT (27–30), SIT (27, 29), TMH (30), CFS (27, 28) and lipid layer thickness (31) deteriorated compared with preoperative status with artificial tear or sodium hyaluronate monotherapy within 1 month after FS-LASIK. In contrast, other studies did not find these changes in TBUT (32), SIT (28), CFS (30) or lipid layer thickness (33). The present study found that OSDI score, SIT, CFS, SRI and lipid layer thickness were significantly worse in the HA group at 1 month after surgery than those in the preoperative period. And although it was not statistically significant, there was a trend toward a decline in BUT following FS-LASIK, which might be due to high variability of BUT and self-healing tendency after FS-LASIK. Regarding corneal nerve, in consistence with prior studies, central corneal sensitivity (27–30, 34) and corneal nerve parameters (34) decreased after FS-LASIK. Several studies have demonstrated that DQS can promote aqueous and lipid secretion, promote mucin secretion, improve tear film stability, promote epithelial repair, inhibit ocular surface inflammation, and relieve subjective discomfort (35, 36), which promises to solve dry eye after FS-LASIK.

In this study, additional DQS significantly reduced subjective symptoms at 1 month postoperatively. Similar symptom improvement was reported in a study of dry eye patients (37). However, Toda et al. reported there was no significant difference between the combination group and the HA group in patients without dry eye before FS-LASIK (19). Different conclusions may stem from different research patients. Unlike previous studies, the present study recruited patients with and without dry eye before FS-LASIK. Consistent with the above-mentioned studies in OSDI score, the present study found significant improvement in the combination group in the dry eye subgroup at 1 month postoperatively and no significant difference in the non-dry eye subgroup. Since the postoperative OSDI score was positively correlated with the preoperative OSDI score, we suppose that HA is effective enough to relieve postoperative dry eye symptoms in the non-dry eye subgroup, but is insufficient to treat dry eye in patients with preoperative dry eye symptoms, for whom additional DQS can further improve dry eye symptoms. Furthermore, by analyzing the three subscales of OSDI, this study found that additional DQS significantly improved the vision-related score, especially in patients with dry eye symptoms before FS-LASIK. The vision-related score is concerned with the discomfort when reading, driving at night, and using a visual display terminal, which affects the quality of life of patients after FS-LASIK. Similarly, Toda et al. also reported that the combined use of DQS and HA after FS-LASIK significantly improved functional visual acuity (FVA) (19), which can reflect visual performance in relation to daily tasks such as computer work, driving, and reading (38). In conclusion, combination therapy with DQS and HA can solve the inconvenience in study and work caused by post-LASIK dry eye.

The additional DQS treatment contributed to the improvement in ocular surface condition in the early postoperative period after FS-LASIK, which could potentially explain the improvement of subjective symptoms. Similar to earlier studies (18), the present study reported that combination therapy with DQS and HA promoted corneal staining, confirming that DQS can promote corneal epithelial repair (39). Interestingly, the combination therapy also had a positive effect on bulbar and limbal redness scores, which might be due to suppression of ocular surface inflammation by DQS (35). Previous study have found that DQS decreases the levels of NF-κB-p65, IL-1β, and TNF-α to inhibit inflammation *via* activation of Erk1/2 and RSK (40). However, in line with the study of Toda, I (19), no significant differences in BUT and SIT were observed between the groups. We speculate that the lack of significance between the groups may be due to large measurement variance and varying speeds of spontaneous remission of dry eye after FS-LASIK.

The lipid layer serves to retard water evaporation from the surface of the open eye and enhance the stability of the tear film. The severity of dry eye symptoms appears to be correlated to lipid layer thickness (41). Additional DQS significantly increased the lipid layer thickness without any changes in meibomian gland status after FS-LASIK in both groups with or without dry eye symptoms before FS-LASIK. Previous animal studies have suggested DQS can improve the number of lipid droplets in meibocytes *in vivo* (42) and stimulate meibocytes to secrete lipid through the P2Y2 receptor *in vitro* (12). The present study found that lipid layer thickness in patients with severe meibomian gland loss was more easily affected by FS-LASIK, and DQS significantly thickened the lipid layer in them while this significance disappeared in patients with good meibomian gland status. We speculate that the meibomian gland with massive loss has low self-recovery ability and reduces the secretion of lipid after surgery, and additional DQS treatment helps meibocytes affected by FS-LASIK produce and release more lipid.

Corneal nerve plays a key role in dry eye after FS-LASIK. In this study, we found that the combination therapy improved corneal sensitivity of non-dry eye subgroup and might make a difference to CNFD and CTBD. A recent study reported that DQS therapy enhanced the number of nerves and beadings, the density of nerves, and nerve tortuosity in dry eye patients with Sjögren’s Syndrome (43). Both of the above studies suggest a possible therapeutic effect of DQS on the corneal nerves. However, DQS did not significantly improve other nerve parameters in the present study possibly because corneal nerve regeneration takes 3–6 months after refractive surgery (44). The mechanism of action of DQS on corneal innervation remains unclear. We speculate that DQS has anti-inflammatory properties and thus reduces the damage to nerve regeneration or that DQS may induce the secretion of nerve growth factor (NGF) by corneal epithelial cells to help nerve regeneration. Further clinical and basic trials are needed to explore the therapeutic effect and potential mechanism of DQS on the corneal nerve.

The present study has several limitations. First, the sample size of the present study was relatively small, resulting in a sample size in stratified analysis. And our study follow-up period of 1 month was relatively short, considering that dry eye after FS-LASIK usually resolved spontaneously within 6–9 months (2). Besides, the study was a cohort study and had intentional selection bias. So more randomized controlled trials with larger sample sizes and longer follow-up time are required to validate our findings. Second, most of the parameters for dry eye such as SIT, NIBUT and TBUT had poor reproducibility, and therefore the assessment of tear film status based on these parameters was not quite accurate. Further studies are needed to focus on components of tear fluid such as mucin or inflammatory factors. In addition, both false-negative and false-positive errors are possible in detecting corneal nerve with ACCMetrics, including the failure to detect thin nerve fibers and the erroneous recognition of other structures such as dendritic cells (45). Third, patients in our study used necessary antibiotics and anti-inflammatory agents after FS-LASIK, which could influence ocular surface status. However, those medications were equally used in the same way in both groups.

To our knowledge, this is the first study to thoroughly evaluate the therapeutic effect of DQS and HA combination therapy, including the quantitative change in lipid layer thickness and subbasal corneal nerve fiber. We found that additional use of DQS significantly improved postoperative lipid layer thickness and potentially promoted subbasal corneal nerve growth after FS-LASIK. The present study is also the first to find the different clinical efficacy of DQS in patients with different preoperative OSDI score levels and different preoperative meibomian gland status. Patients with dry eye symptoms or patients with worse meibomian gland status are more likely to benefit from additional usage of DQS. The findings of our study provide an evidence for a novel treatment strategy involving the additional DQS for the postoperative management of patients with dry eye symptoms or risk factors for dry eye.

In conclusion, combination therapy with DQS and sodium hyaluronate was more effective than monotherapy with sodium hyaluronate for dry eye after FS-LASIK, especially in patients who had dry eye symptoms or massive meibomian gland loss.

## Data availability statement

The raw data supporting the conclusions of this article will be made available by the authors, without undue reservation.

## Ethics statement

The studies involving human participants were reviewed and approved by the Ethics Committee of Peking Union Medical College Hospital. The patients/participants provided their written informed consent to participate in this study.

## Author contributions

TW and YD performed the research, analyzed the data, and drafted the manuscript and manuscript revision. YL conceived of the study, administered and coordinated the research, and revised the manuscript. All authors contributed to the article and approved the submitted version.

## Conflict of interest

The authors declare that the research was conducted in the absence of any commercial or financial relationships that could be construed as a potential conflict of interest.

## Publisher’s note

All claims expressed in this article are solely those of the authors and do not necessarily represent those of their affiliated organizations, or those of the publisher, the editors and the reviewers. Any product that may be evaluated in this article, or claim that may be made by its manufacturer, is not guaranteed or endorsed by the publisher.
