# Supplementary material for: Combination therapy with 3% diquafosol tetrasodium ophthalmic solution and sodium hyaluronate: an effective therapy for patients with dry eye after femtosecond laser-assisted in situ keratomileusis
Source: Front Med (Lausanne). 2023 Apr 20;10:1160499. doi: 10.3389/fmed.2023.1160499 (PMC10157480; doi:10.3389/fmed.2023.1160499)
Supplement: Supplementary file 1 [file Table_1.docx]

**SUPPLEMENTARY TABLE 1. Postoperative alterations of dry eye parameters, meibomian gland parameters and corneal nerve parameters in the combination group and the HA group**

|  | Preoperative-postoperative 1 week | | |  | Preoperative-postoperative 1 month | | |  | Postoperative 1 week-postoperative 1 month | | |
| --- | --- | --- | --- | --- | --- | --- | --- | --- | --- | --- | --- |
|  | DQS+HA | HA | *P* |  | DQS+HA | HA | *P* |  | DQS+HA | HA | *P* |
| Subjective Symptoms | | | | | | | | | | | |
| ΔOSDI | 9.52±16.21 | 20.33±27.99 | 0.143 |  | -0.58±14.05 | 10.33±19.92 | 0.053 |  | -10.10±16.85 | -10.00±14.78 | 0.820 |
| ΔOcular symptom score | 13.25±16.88 | 19.25±27.21 | 0.231 |  | 4.00±10.95 | 9.25±23.24 | 0.068 |  | -9.25±19.14 | -10.00±13.18 | 0.779 |
| ΔVision-related score | 4.79±30.42 | 27.81±42.14 | 0.055 |  | -9.58±34.97 | 14.27±31.50 | 0.017* |  | -14.38±22.96 | -13.54±25.06 | 0.738 |
| ΔEnvironmental score | 8.33±19.69 | 11.40±29.10 | 0.705 |  | 4.17±14.75 | 6.67±20.87 | 0.665 |  | -4.61±16.07 | -3.95±15.68 | 0.899 |
| Ocular Surface Parameters | | | | | | | | | | | |
| ΔTMH (mm) | -0.01±0.10 | -0.01±0.07 | 0.630 |  | 0.01±0.11 | 0.00±0.10 | 0.509 |  | 0.02±0.007 | 0.02±0.10 | 0.586 |
| ΔSIT (mm) | -4.2±9.8 | -4.8±8.8 | 0.809 |  | -2.3±9.2 | -4.5±7.7 | 0.245 |  | 1.9±8.6 | 0.3±9.4 | 0.537 |
| ΔCFS | 0.5±2.3 | 1.4±1.9 | 0.018* |  | 0.6±2.1 | 1.1±1.9 | 0.139 |  | 0.2±2.2 | -0.4±2.4 | 0.459 |
| ΔNIBUT-First (s) | 0.70±8.79 | -1.33±9.40 | 0.321 |  | -1.79±7.20 | -1.30±8.04 | 0.772 |  | -2.49±8.72 | 0.04±8.69 | 0.145 |
| ΔNIBUT-Ave (s) | -0.02±8.02 | -0.50±9.45 | 0.807 |  | -1.37±8.86 | -0.98±7.55 | 0.834 |  | -1.35±8.67 | -0.48±9.67 | 0.674 |
| ΔTBUT (s) | -0.8±4.4 | -0.5±4.9 | 0.900 |  | -1.3±3.6 | -1.2±3.8 | 0.907 |  | -0.5±3.3 | -0.7±3.1 | 0.835 |
| ΔBulbar redness score | -0.05±0.16 | 0.03±0.17 | 0.021* |  | -0.02±0.24 | 0.03±0.19 | 0.525 |  | 0.03±0.18 | 0.00±0.21 | 0.474 |
| ΔLimbal redness score | -0.09±0.14 | -0.01±0.13 | 0.009** |  | -0.03±0.22 | 0.02±0.16 | 0.580 |  | 0.06±0.15 | 0.02±0.13 | 0.112 |
| ΔSRI |  |  |  |  | 0.13±0.25 | 0.16±0.23 | 0.633 |  |  |  |  |
| Meibomian Gland Parameters | | | | | | | | | | | |
| ΔLipid layer grade | -0.3±1.5 | -0.9±1.6 | 0.038* |  | 0.3±1.5 | -0.9±1.7 | 0.003** |  | 0.5±1.4 | 0.0±1.2 | 0.212 |
| ΔMeiboscore | 0.1±0.5 | 0.1±0.5 | 1.000 |  | 0.2±0.5 | 0.1±0.4 | 0.965 |  | 0.1±0.5 | 0.1±0.5 | 0.753 |
| ΔLid margin abnormality | 0.0±0.7 | 0.1±0.6 | 0.408 |  | 0.1±0.8 | 0.3±0.8 | 0.399 |  | 0.2±0.7 | 0.2±0.8 | 0.982 |
| Corneal Nerve Parameters | | | | | | | | | | | |
| ΔCorneal sensitivity (mm) | -45.9±19.3 | -47.4±14.6 | 0.918 |  | -37.9±20.5 | -41.8±18.6 | 0.559 |  | 8.0±22.1 | 5.6±17.7 | 0.353 |
| ΔCNFD (/mm^2^) | -19.4±7.4 | -19.7±7.3 | 0.835 |  | -19.2±7.4 | -19.8±7.1 | 0.712 |  | 0.2±2.4 | -0.1±0.9 | 0.971 |
| ΔCNBD (/mm^2^) | -24.5±14.2 | -21.2±15.6 | 0.182 |  | -24.2±14.2 | -21.2±15.3 | 0.228 |  | 0.3±2.1 | 0.1±1.2 | 0.530 |
| ΔCNFL (mm/mm^2^) | -10.5±3.2 | -10.4±2.9 | 0.886 |  | -10.5±3.1 | -10.6±2.8 | 0.929 |  | 0.0±1.8 | -0.2±1.3 | 0.664 |
| ΔCTBD (/mm^2^) | -32.3±18.9 | -32.1±22.6 | 0.704 |  | -33.9±20.1 | -32.4±21.6 | 0.576 |  | -1.5±7.8 | -0.4±5.0 | 0.812 |
| ΔCNFA (mm^2^/mm^2^) | -0.0039±0.0019 | -0.0036±0.0021 | 0.283 |  | -0.0040±0.0019 | -0.0039±0.0018 | 0.780 |  | -0.0001±0.0014 | -0.0002±0.0010 | 0.595 |
| ΔCNFW(mm/mm^2^) | 0.006±0.006 | 0.008±0.003 | 0.138 |  | 0.006±0.004 | 0.007±0.004 | 0.141 |  | 0.000±0.007 | -0.001±0.004 | 0.985 |
| ΔCNFrD | -0.25±0.11 | -0.24±0.06 | 0.795 |  | -0.25±0.09 | -0.25±0.06 | 0.876 |  | 0.00±0.11 | -0.01±0.07 | 0.583 |

Data are presented as means±standard deviation. OSDI, ocular surface disease index; TMH, tear meniscus height; SIT, Schirmer I test; CFS, corneal fluorescein staining score; NIBUT-First, first non-invasive tear breakup time; NIBUT-Ave, average non-invasive tear breakup time; TBUT, tear breakup time; SRI, surface regularity index; CNFD, nerve fiber density; CNBD, nerve branch density; CNFL, nerve fiber length; CTBD, nerve fiber total branch density; CNFA, nerve fiber area; CNFW, nerve fiber width; CNFrD, nerve fiber fractal dimension. ^*^*P*<0.05 and ***P*<0.01 between the combination group and the HA group by independent samples t-test or Mann-Whitney U test. DQS, diquafosol tetrasodium; HA, sodium hyaluronate; DQS+HA, combination of diquafosol tetrasodium and sodium hyaluronate.

**SUPPELEMENTARY TABLE 2. Changes in LLG in low meiboscore subgroup and high meiboscore subgroup after FS-LASIK**

|  | Low meiboscore subgroup | | |  | High meiboscore subgroup | | |
| --- | --- | --- | --- | --- | --- | --- | --- |
|  | DQS+HA | HA | *P* |  | DQS+HA | HA | *P* |
| Preoperative LLG | 4.6±1.3 | 4.5±1.4 | 0.873 |  | 4.4±1.3 | 4.3±1.3 | 0.578 |
| LLG_1w | 3.9±1.2 | 4.2±1.3 | 0.423 |  | 4.4±1.1 | 3.1±1.1^††^ | <0.001^***^ |
| LLG_1m | 5.1±0.8 | 4.6±1.2 | 0.371 |  | 4.5±0.9 | 2.9±1.1^††^ | <0.001^***^ |
| ΔLLG(0-1w) | -0.7±1.5 | -0.3±2.0 | 0.664 |  | 0.0±1.4 | -1.2±1.3 | 0.002^**^ |
| ΔLLG(0-1m) | 0.4±1.7 | 0.1±1.8 | 0.598 |  | 0.1±1.5 | -1.3±1.5 | 0.002^**^ |
| ΔLLG(1w-1m) | 1.1±1.5 | 0.4±1.4 | 0.280 |  | 0.1±1.2 | -0.1±1.1 | 0.658 |

Data are presented as means±standard deviation. The sample sizes of the four groups were 16, 12, 24, and 28 eyes, respectively. LLG, lipid layer grade; LLG_1w, lipid layer grade at postoperative 1 week; LLG_1m, lipid layer grade at postoperative 1 month; ΔLLG(0-1w), the alteration of LLG from preoperative to postoperative 1 week; ΔLLG(0-1m), the alteration of LLG from preoperative to postoperative 1 month; ΔLLG(1w-1m), the alteration of LLG from postoperative 1 week to postoperative 1 month. ^*^*P*<0.05, ^**^*P*<0.01, and ^***^*P*<0.001 between the combination group and the HA group by independent samples t-test or Mann-Whitney U test. ^†^*P*<0.05 and ^††^*P*<0.01 between preoperative visit and postoperative visits in the HA group by one-way repeated measures ANOVA or Friedman test. DQS, diquafosol tetrasodium; HA, sodium hyaluronate; DQS+HA, combination of diquafosol tetrasodium and sodium hyaluronate.
